# Effect of aerial application of adjuvants on pepper defoliant droplet deposition and efficacy of defoliation sprayed by unmanned aerial vehicles

**DOI:** 10.3389/fpls.2022.917462

**Published:** 2022-09-08

**Authors:** Yapeng Liu, Qinggang Xiao, Xiaoqiang Han, Muhammad Zeeshan, Zhihao Fang, Zechen Dou

**Affiliations:** Key Laboratory of Oasis Agricultural Pest Management and Plant Protection Utilization, College of Agriculture, Shihezi University, Shihezi, China

**Keywords:** processing pepper, defoliant, droplet deposition, aviation spray adjuvants, unmanned aerial vehicle (UAV)

## Abstract

Defoliant spraying is an important aspect of the mechanized processing of pepper harvesting. Complete and uniform spraying of defoliant could improve the quality of defoliation and reduce the impurity content in processing pepper. In this study, we assessed the effect of aerial spraying of adjuvants on physicochemical properties of defoliant solution and droplet deposition when using an unmanned aerial vehicle (UAV) for defoliation spraying. The results showed that Puliwang was a better aerial spray adjuvant suitable for spraying defoliants for processing pepper using UAVs, with a higher defoliation rate and better droplet deposition. Although the YS-20 adjuvant had a higher droplet deposition amount (0.72 μg/cm^2^) in the middle layer, its performance was poor in droplet size, density, and coverage. The size and density of the droplets added with the Manniu were basically the same as the Puliwang, even the distribution uniformity was better (the CV of the upper canopy layer was only 33.6%), but the coverage rate was poor. In the treatment with AS-901N, there was no marked increase in droplet size, so evaporation and drift were not improved, eventually resulting in a lower defoliation rate. Puliwang had the highest comprehensive score, followed by AS-910N, YS-20, and Manniu.

## Introduction

Peppers (*Capsicum annuum* L.), with a variety of nutrients, are widely used in cosmetics, food additives, and as an important pharmaceutical and industrial raw material (Baenas et al., 2019). Xinjiang is an important production and processing area for peppers in China where the land, sunlight, and heat resources are abundant. Peppers from this region are highly reputed and have market competitiveness in domestic and foreign markets (Chai et al., 2020). In 2018, the area of processing pepper in Xinjiang was 3.67 × 10^4^ hm^2^, and the annual yield contributed to 20% of the country's total production which was 25 × 10^4^ t. At the later stage of the plant's growth, defoliants are applied to make it ready for harvesting by shedding its leaves. As the stalks and branches of the plants are extremely fragile, the traditional boom sprayer can damage the crop by crushing the leaves as well as the mature fruit which will ultimately affect the yield and quality of the pepper (Xiao et al., 2020).

In recent years, unmanned aerial vehicles (UAVs) have made great strides in agriculture. Their spraying operations increase the deposition of pesticides on target crops and avoid physical damage to crops during ground equipment operations (Yuan et al., 2018). The distancing of humans and machines from the crops avoids pesticide poisoning and greatly improves the efficiency of pesticide spraying and the utilization rate of pesticides (Yan et al., 2021a). UAVs also have low levels of water consumption, low dilution ratios, high working heights, fast flight speeds, and high atomization abilities. UAVs have been widely used in crops such as wheat (Yan et al., 2021b), rice (Chen et al., 2020a), corn (Hussain et al., 2022), grapes (Matese and Di Gennaro, 2018), citrus (Tang et al., 2018), and cotton (Lou et al., 2018). The addition of aerial spray with adjuvants efficiently solves the drift and loss of pesticide droplets during the spraying by UAVs and improves the utilization rate of pesticides.

Extant research on the effect of UAV spraying has mainly focused on fertilization, pesticide application, and nutritional analysis (Qiu et al., 2021; Xu et al., 2021; Hafeez et al., 2022). However, UAV spraying is greatly affected by the environment, which makes it easy for the droplets to drift and evaporate, resulting in reduced pesticide utilization and environmental concerns (Wang et al., 2020). Adding adjuvants to the aerial spray is an effective method to solve the drift and loss of pesticide droplets during UAV spraying (Chen et al., 2021). Xiao et al. (2019) studied the effects of five aerial application adjuvants on droplet deposition of cotton defoliation and found that vegetable oil adjuvants had a better effect. However, limited research is available on the effects of aerial spray adjuvants on pepper defoliant processing. In this study, we studied “Honglong 18” pepper as the test material using T16 UAV as the spraying equipment and examined the efficacy of four kinds of aerial application adjuvants. The physicochemical properties of the defoliant solution were studied through laboratory experiments, and the deposition characteristics were further analyzed to assess the defoliation effect of the defoliant droplets sprayed by UAVs through field experiments. Through this study, we aim to provide theoretical guidance for the operation of the UAV spraying process in pepper defoliants.

## Materials and methods

### Materials

The pepper defoliant (18% glufosinate ammonium soluble concentrate) was produced by Beijing Zhongnong Honglu Technology Development Co., Ltd., Beijing, China. The tested adjuvants were YS-20 (improved vegetable oil adjuvant, Anyang Quanfeng Biotechnology Co., Ltd., Anyang, China), Manniu (vegetable oil adjuvant, Qingdao Rishengyuan Crop Nutrition Co., Ltd., Qingdao, China), Puliwang (vegetable oil adjuvant, Oro Agri. International Co., Ltd., Palmela, Portugal), and AS-910N (improved vegetable oil adjuvant Momentive Performance Materials Inc., New York, USA). Allura Red (85%) was used as a droplet tracer (Zhejiang Gigagold Pigment Technology Co., Ltd., Wenzhou, China) and ethephon aqueous solutions (40%) were used as a ripening agent (Jiangsu Anpon Electrochemical Co., Ltd., Changzhou, China).

The aviation platform used was the T16 UAV (SZ DJI Technology Co., Ltd., Shenzhen, China). The UAV was equipped with RTK/GNSS precise positioning system, and its spraying system included a little water pump, pipeline, nozzles (8 XR11001VS, located directly below the rotor), and electronic control valve. T16 UAV has six rotors with a 16.0 L water tank and a payload of 15 kg. The flight height was 2.0 m and flight speed of 5.0 m/s with a spray width of 5 m and spraying volume of 15.0 L/hm^2^.

### Treatments

There were five treatments in the experiment (Table 1). Treatments 1, 2, 3, and 4 were added with YS-20, Manniu, Puliwang, and AS-910N in the dosage of 225 g/hm^2^. Treatment 5 was the control (CK) without adjuvant. In addition, 1,875 g/hm^2^ of pepper defoliant, 300 g/hm^2^ of Allura red, and 900 g/hm^2^ of 40% ethephon aqueous solution were added to each treatment.

**Table 1:** Test treatment design.

**Treatment**	**Adjuvants**	**Dosage of adjuvants** **(g/hm^2^)**	**Defoliant** **(g/hm^2^)**	**Ethephon** **(g/hm^2^)**	**Spraying volume** **(L/hm^2^)**
1	YS-20	225	1,875	900	15
2	Manniu	225	1,875	900	15
3	Puliwang	225	1,875	900	15
4	AS-910N	225	1,875	900	15
5	/	/	1,875	900	15

### Determination of physicochemical properties of pesticide solution

#### Surface tension

The surface tension was measured using the ST-1510 automatic interfacial tension meter (Xuxin Instrument Equipment Co., Ltd., Beijing, China) adopting the ring method according to GB/T 6541-1986, 10s after pesticide solution preparation. Each treatment was measured three times.

#### Dynamic viscosity

Kinematic viscosity of pesticide solution was measured by an electronic analytical balance [Sartorius Scientific Instruments (Beijing) Co., Ltd, Beijing, China], calculated by Equation 1 (Gao et al., 2021). Each treatment was measured three times.


(1) $$\begin{array}{r} \eta =\rho \times \left(v_{k}\times 0.00947\right) \end{array}$$

where η is the dynamic viscosity (mPa·s), ρ is the density (g/mL), *v*_*k*_ is the kinematic viscosity, and 0.00947 is the instrument constant for this viscometer (mm^2^/s^2^), given by the manufacturer.

#### Contact angle

Fresh pepper leaves (2 × 2 cm, avoiding leaf veins, disease spots, etc.) were fixed the on the slide, and 2 μL pesticide solution (Table 1) was dropped on the leaves, respectively. The contact angle was recorded by drop shape analyzer DSA100 (KRUSS, Hamburg, Germany). Each treatment was recorded for three replicates.

#### Spreading ratio

Fresh pepper leaves (2 × 2 cm, avoiding leaf veins, disease spots, etc.) were placed the on the worktable of DP74 stereomicroscope (Olympus Co., Ltd., Japan), with a magnification of 10 times. About 2 μL of pesticide solution (Table 1) was dropped on the leaves and the spreading area of the droplet was recorded. The spreading ratio was calculated by Equation 2. Each treatment was recorded for three replicates.


(2) $$\begin{array}{r} R=\left(S_{t}/S_{0}\right)\times 100\% \end{array}$$

where *R* is the spreading ratio, *S*_*t*_ is the spreading area at *t* s, and *S*_0_ is the initial area.

#### Field and conditions

The experiment was carried out in the Beiquan town of Xinjiang production and construction crops (44°23'11 “N, 86°6'11” E), Shihezi, Xinjiang, China, in 2019. The experimental field was fertilized to a moderate level and had planted peppers for 2 years. The peppers (Honglong 18) were sown on 13 April 2019 with a wide film model having six lines (10 ± 66 cm) and 210,000 plants/hm^2^ (the actual number of plants was 12,070 plants/667 m^2^), and were irrigated by drip irrigation under the film. The defoliant was sprayed from 10 am to 12 am on 12 September 2019, from an average height of 0.88 m. The average wind speed was 2.06 m/s with relative humidity of 36.90% and temperature at 22.13°C (Kestrel 5500, Nielsen-Kellerman, Boothwyn, USA).

**Figure 1:**
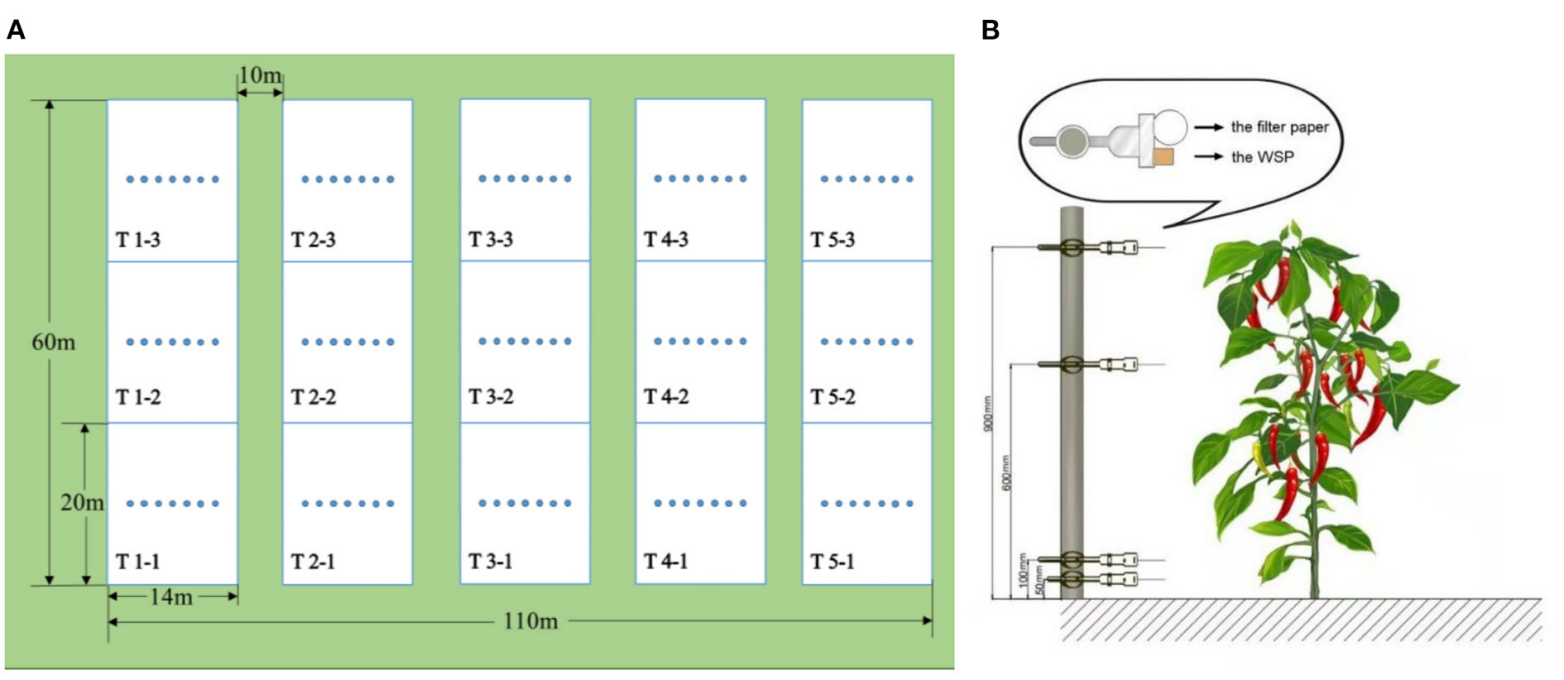
**(A)** The experimental layout of each treatment and **(B)** the placement of the WSP and filter papers at each sampling position within the processing pepper.

There were three replicates of 2,700 m^2^ each in every treatment, with a 10 m buffer area between each treatment (Figure 1A). A droplet information collection belt was set in the middle of each repetition and was perpendicular to the UAV route. Seven droplet information collection points were arranged in an orderly manner on the belt with a spacing of 0.5 m. A metal stick was inserted at the point, and a water sensitive paper (WSP, 26 × 76 mm) and a filter paper (*d* = 70 mm) were fixed at a distance of 900, 600, 100, and 50 mm from the ground through double-sided clips, in line with the upper layer, middle layer, the lower layer of pepper canopy and ground (Figure 1B). After spraying, we waited for the WSP and filter paper to dry slightly, then marked and collected them before taking them back to the lab for analysis.

### Determination of droplet deposition

#### Droplet information

Droplet information, including droplet size, droplet density, and droplet coverage, was obtained by first scanning the collected WSP with a FileScan2500 scanner (Shanghai Zhongjing Technology Co., Ltd., Shanghai, China) at grayscale and 600 dpi parameters and then analyzing it with Image J 1.38X software (National Institutes of Health).

#### Droplet deposition

The droplet deposition was obtained by measuring the Allura red content on the filter paper. About 5 ml of distilled water was added to each zip lock bag with filter paper and washed with a small shaker for 10 min, then centrifuged at 4,000 rpm for 5 min (Eppendorf 5417R Centrifuge, Eppendorf Co., Ltd., Hamburg, Germany). The absorbance value (Y_i_) was determined using the Infinite 200Pro ELISA instrument (Tecan, Meilen, Switzerland) at 510 nm. The Y_i_ was then converted to mass concentration and X_i_ according to the linear regression equation of the Allura red standard solution (Y = 0.0238X + 0.0431, *R*^2^ = 0.997). The deposition amount per unit area was calculated using Equation 3.


(3) $$\begin{array}{r} A=\frac{X_{i}\times V}{S} \end{array}$$

where *A* is droplet deposition per unit area, *X*_*i*_ is the mass concentration of eluent, *V* is the volume of the added eluent, *S* is the area of droplet collector.

#### Droplet distribution uniformity

The uniformity of droplet distribution was calculated by the coefficient of variation (*CV*) of the same canopy deposition of processed peppers, and calculated using Equations 4, 5 (Lou et al., 2018).


(4) $$\begin{array}{r} s\;=\sqrt{\frac{1}{n-1}\overset{n}{\underset{i=1}{\sum }}{\left(X_{i}-X\right)}^{2}} \end{array}$$

(5) $$\begin{array}{r} CV=\frac{s}{X} \end{array}$$

where *s* is the variance, *CV* is the coefficient of variation, *X*_*i*_ is the droplet information (droplet density, DV_50_ and coverage) of each droplet captured card, *X* is the droplet information (droplet density, DV_50_ and coverage rate) of different parts of the pepper plant coverage, *n* is the total number of droplet collection cards in different parts of the pepper plant.

#### Spraying penetration

The spraying penetration was measured using the ratio of the deposition amount of the upper layer and lower layer of pepper canopy, as shown in Equation 6.


(6) $$\begin{array}{r} P=\frac{A_{d}}{A_{u}}\times 100\% \end{array}$$

where *P* is the spraying penetration rate, *A*_*d*_ is the deposition amount in the lower layer of the processing pepper canopy, and *A*_*u*_ is the deposition amount in the upper layer of the processing pepper canopy.

#### Effective droplet deposition rate

After all the sample concentration values were measured, the deposition amount and deposition rate of droplets at each sampling point were calculated according to ISO22866 standard (ISO/TC 23/SC, 2005). And it was calculated using Equations 7–9 (Chen et al., 2020b).


(7) $$\begin{array}{r} D_{s}=\frac{F}{V\times I}\times 1.67 \end{array}$$

(8) $$\begin{array}{r} D_{d}=\frac{C_{e}\times V}{C_{s}\times A} \end{array}$$

(9) $$\begin{array}{r} R=\frac{D_{d}}{D_{S}}\times 100\% \end{array}$$

where *D*_*s*_ is the deposition amount per unit area (μL/cm^2^), *V* is the flight speed (m/s), *I* is the spraying interval (m), *F* is the spraying flow rate of the UAV (L/min), 1.67 is a constant. *D*_*d*_ is the deposition amount per unit area (μL/cm^2^), *C*_*e*_ is the concentration of the eluent (μg/mL), *V* is the volume of the eluent (mL); *C*_*S*_ is the concentration of the tracer (g/L); *A* is deposition sampling area (cm^2^). *R* is the deposition rate.

#### Defoliation rate

Three points with consistency and representativeness were randomly selected in each replicate area. Then 10 consecutive pepper plants were selected from each point to investigate the total number of leaves before spraying. They were re-investigated 3, 5, 7, 9, and 12 d after spraying and the defoliation rate was calculated using Equation 10.


(10) $$\begin{array}{r} R_{d}=\frac{S_{1}-S_{2}}{S_{1}}\times 100\% \end{array}$$

where *S*_1_ is the number of leaves investigated before spraying; *S*_2_ is the number of leaves investigated after spraying.

#### Yield

At the time of harvesting the peppers (24 September 2019), three sampling sites were selected for each treatment, 15 consecutive pepper plants were selected from each site, and all their fruits were collected and the fresh fruit were weighed. After 30 days of air-drying, the harvested peppers were weighed to estimate the yield using Equation 11.


(11) $$\begin{array}{r} Y=Y\times \frac{12070}{15} \end{array}$$

where *Y* is the theoretical yield (kg/667 m^2^), *Y* is the average of fresh (dry) weight of peppers from three sampling points in each replicate (kg). With an harvest of 14,200 the planting density of the pepper field was 12,070 plants per 667 m^2^.

#### Data analysis

All data were analyzed by OriginPro 2022b (Origin Lab, Northampton, MA, USA) and SPSS 22 (SPSS Inc., an IBM Company, Chicago, IL, USA) statistical software. Duncan's new multiple range test was selected to test the significance of differences at the level of *P* < 0.05.

## Results and discussion

### Effect of aerial application adjuvants on dynamic viscosity and surface tension

**Figure 2:**
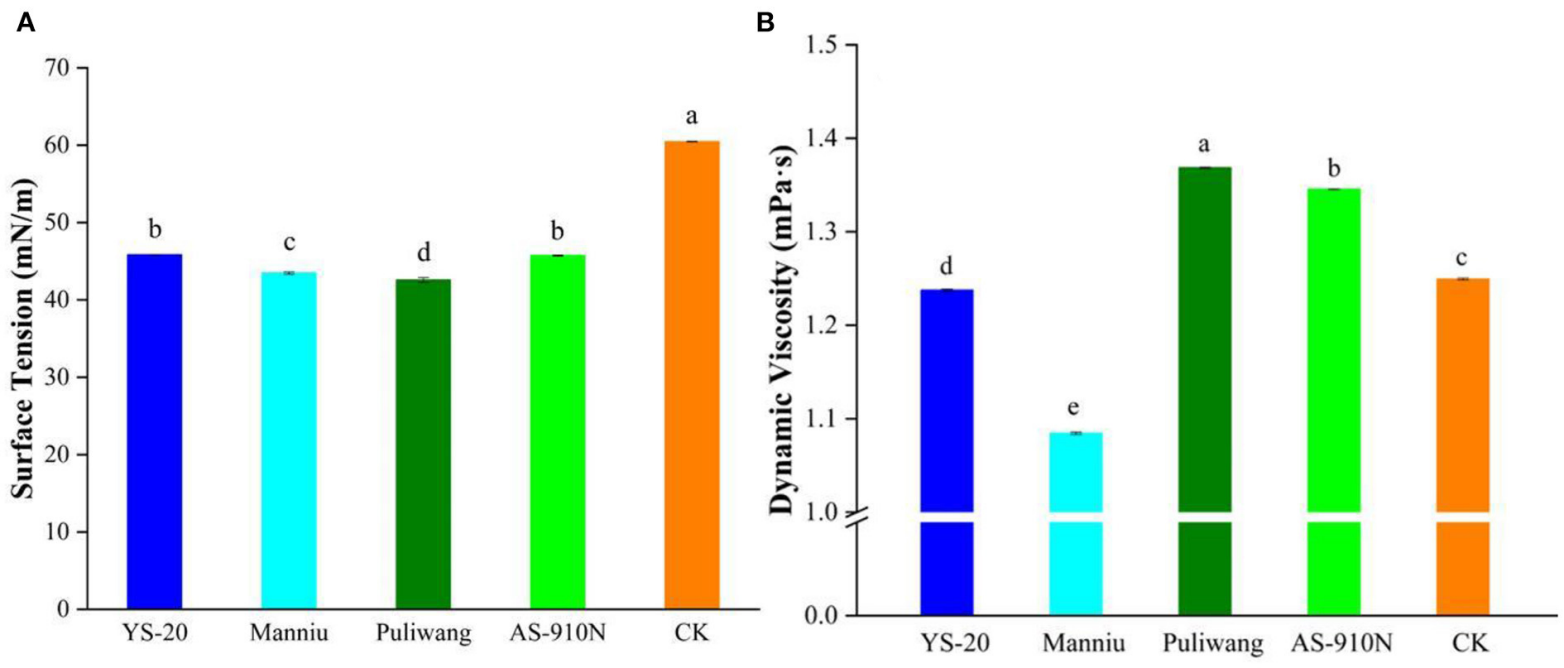
Effect of aerial spray with adjuvants on surface tension **(A)** and dynamic viscosity **(B)**. ^a−e^They represent the results of a significant difference analysis by Duncan's new multiple range test at the level of *P* < 0.05. Values followed by the same letter do not differ statistically.

Reducing the surface tension of the pesticide solution can enhance the wetting performance and spreading ability of the spraying solution on the leaves. At 10 s, the surface tension of the YS-20, Manniu, Puliwang, AS910N, and CK was 45.83, 43.47, 42.57, 45.73, and 60.47 mN/m, respectively (Figure 2A). All four kinds of aerial spray adjuvants significantly reduced the surface tension of the pesticide solution to <46 mN/m. Compared with the surface tension of CK, Puliwang had the best effect on reducing the surface tension, which decreased by 29.6%. Liquid viscosity affects the atomization performance of the nozzle and also the number of satellite droplets, coalescence, viscosity dissipation in the collision process, and the spread of droplets on the leaves (Brenn and Kolobatic, 2006). As shown in Figure 2B, Puliwang and AS-910N could increase the viscosity to 1.37 and 1.34 mPa·s, respectively. The Manniu reduced the viscosity, while YS-20 had no effect on viscosity. The increase of viscosity is helpful to the deposition of droplets on leaves and avoids the bounce of droplets (Song et al., 2019). Pepper leaves are hydrophilic leaves. Increasing the viscosity of droplets, therefore, is conducive to the attachment of droplets on the leaves and improves the efficacy.

### Effect of aerial application of adjuvants on contact angle

**Figure 3:**
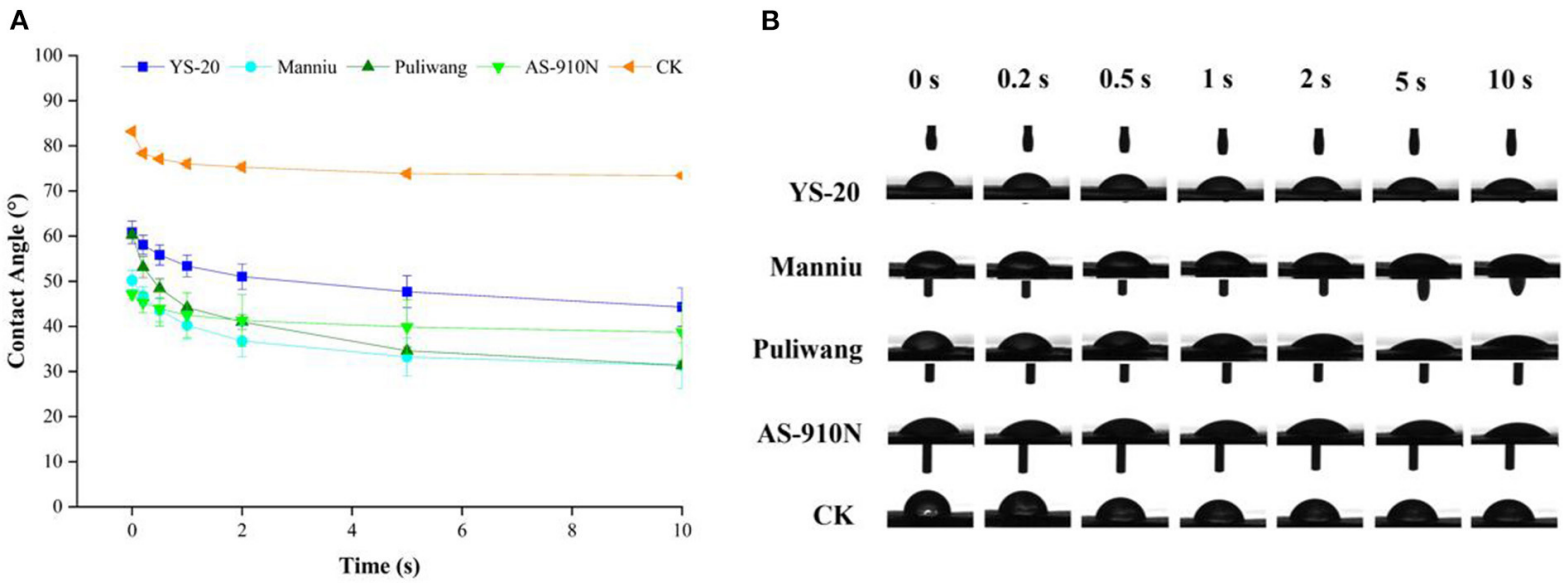
Effect of aerial spray with adjuvants on contact angle line graph **(A)** and experimental images **(B)**.

Adjuvants showed a significant effect on the contact angle of the droplet in the initial state (Figure 3). At 0 s, the contact angle without adjuvant was 83.20°, while that of YS-20, Manniu, Puliwang, and AS-910N were 60.85°, 50.22°, 60.26°, and 47.12°, respectively. The contact angle decreased rapidly within 10 s and gradually leveled off. After 5 s, the rate of contact angle slows down and tends to be stable. This indicated that the adjuvants could spread the spray solution more easily on pepper leaves, which is beneficial for the absorption of the defoliant. The adjuvant affected the contact angle by moderating the surface tension. In general, the contact angle of the leaf surface of the same crop will decrease with a decrease in surface tension (Lan et al., 2021). Xu et al. (2011) found that increasing viscosity and reducing surface tension were two main methods used to increase pesticide retention on superhydrophobic rice leaf surfaces. This was also consistent with our results, where after adding adjuvants the surface tension and contact angle of the droplets displayed the same trend. Our results showed that the surface tension and the contact angle of the pesticide solution on the pepper leaves were reduced, but the effect was different, which was based on the specific adjuvant used.

### Effect of aerial spray with adjuvants on spreading ratio

**Figure 4:**
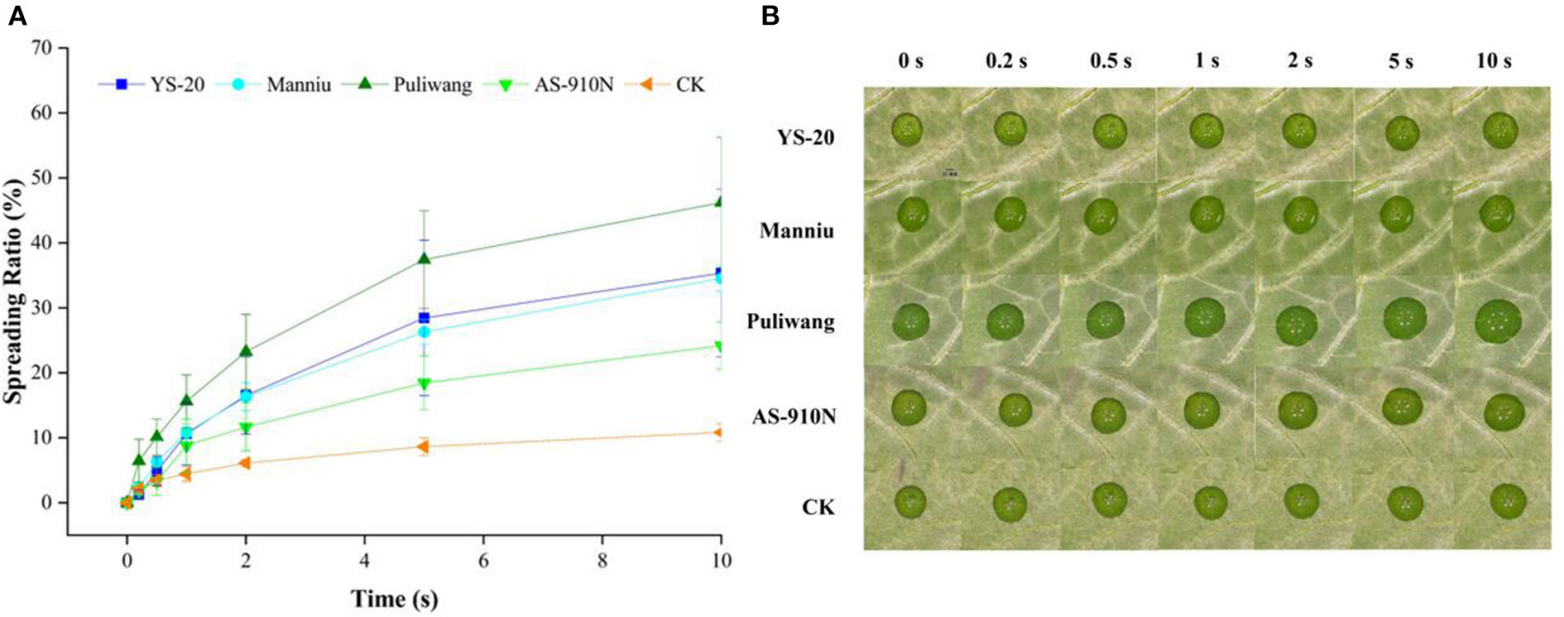
Effect of aerial spray with adjuvants on spreading ratio line graph **(A)** and experimental images **(B)**.

The spreading of fluids over solid substrates is of great importance to pesticide applications, including defoliants. The wetting and spreading of pesticides on the leaf surface are closely related to the combination of the leaf surface and the physicochemical properties of the pesticide solution. We found that adding aerial applications with adjuvants could increase the spreading ratio of defoliant droplets on the surface of pepper leaves (Figure 4). At 10 s, the spreading ratio of YS-20, Manniu, Puliwang, and AS-910N was 35.35, 34.54, 46.21, and 24.17%, respectively, which was significantly higher than that of CK (10.81%). This result was consistent with the analysis results of the contact angle. Different types of adjuvants can improve various aspects of spray dilution performance. Beacham et al. (2009) found that organosilicon adjuvants have a very prominent effect on improving the wetting of pesticide droplets on the leaf surface. However, when defoliants were used, ethephon, a strong acid ripening agent, needs to be added, which greatly destroys the stability of organosilicon adjuvants. Vegetable oil and modified vegetable oil adjuvants have been popular in recent years because of their wide tolerance. Zhou et al. (2017) found that modified seed oil slows down the evaporation rate of droplets on waxy leaves. Our experiments showed that four vegetable oil adjuvants could also effectively improve the spreading rate of defoliant droplets on pepper leaves and improve the defoliation effect.

### Effect of aerial applications with adjuvants on the droplet size sprayed by UAV

Many factors, including adjuvants, pesticide formulations, and nozzles affect spray droplet size (Klein et al., 2009). The droplet size is one of the important indicators to evaluate the spraying quality when using UAVs. As shown in Table 2, the average droplet size of the treatments with adjuvants was significantly larger than that of those without adjuvants. The droplet sizes of the four adjuvants were also different. In the upper layer, the DV_10_ and DV_50_ of YS-20, Manniu, and Puliwang were larger than AS910N and CK, the DV_90_ showed no difference. In the middle and lower layers, the DV_10_ and DV_50_ of Puliwang were larger than others, but not significant. On the ground, the DV_10_ and DV_50_ of the treatments with or without adjuvants had no difference. Overall, the DV_50_ in the upper, middle, and lower layers, and ground with Puliwang was 402 ± 22.5, 377 ± 24, 365 ± 27.8, and 355 ± 28.8 μm, respectively, which was higher than other adjuvant augmented treatments. Although there is no specific droplet size range that is likely to drift under all conditions, droplets with diameters <100 μm are considered highly draftable (Nuyttens et al., 2014; Ferguson et al., 2016).

**Table 2:** Effect of aerial application of adjuvants on the droplet size sprayed by UAVs.

**Pepper canopy**	**Treatment**	**Droplet size (**μ**m)**		
		**DV_10_**	**DV_50_**	**DV_90_**
Upper layer	YS-20	238 ± 11.7 a	373 ± 30.4 ab	553 ± 31.5 a
	Manniu	219 ± 12.3 a	397 ± 29.6 a	650 ± 38.5 a
	Puliwang	226 ± 3.8 a	402 ± 22.5 a	628 ± 34.4 a
	AS-910N	171 ± 12.7 b	320 ± 15.3 b	523 ± 35.5 a
	CK	193 ± 17.1 b	348 ± 53.9 ab	571 ± 129 a
Middle layer	YS-20	220 ± 17.8 ab	350 ± 58.2 ab	495 ± 120.4 ab
	Manniu	208 ± 25.2 ab	368 ± 47.3 a	522 ± 83.8 ab
	Puliwang	227 ± 4.5 a	377 ± 24 a	553 ± 59.9 a
	AS-910N	153 ± 27.9 c	275 ± 34.6 b	402 ± 68.5 ab
	CK	184 ± 16.2 bc	288 ± 19.8 b	395 ± 32 b
Lower layer	YS-20	204 ± 19.4 b	311 ± 17.9 b	405 ± 37.7 b
	Manniu	205 ± 13 b	317 ± 28.3 ab	432 ± 53.4 ab
	Puliwang	235 ± 16.1 a	365 ± 27.8 a	509 ± 60.4 a
	AS-910N	179 ± 7.3 bc	292 ± 42 b	373 ± 67.7 b
	CK	163 ± 9.1 c	271 ± 18.9 b	367 ± 28.2 b
Ground	YS-20	228 ± 28.1 a	328 ± 54.5 a	437 ± 42.1 a
	Manniu	205 ± 33.1 abc	330 ± 40.9 a	446 ± 63.6 a
	Puliwang	225 ± 21.9 ab	355 ± 28.8 a	485 ± 63.7 a
	AS-910N	163 ± 43.2 c	268 ± 60.6 a	370 ± 94.6 a
	CK	167 ± 22.9 bc	291 ± 43.4 a	391 ± 90.1 a

Values followed by the same letter in the column do not differ statistically (p < 0.05).

Matthews et al. reported that the optimum droplet size for herbicide spraying is 250 μm, while for fungicide, the particle size should be maintained at 50–150 μm (Matthews and Thomas, 2000). In this study, the droplet size in the treatment after adding Puliwang was between 350 and 450 μm, significantly higher than other treatments; the final defoliation rate was similarly the highest. It demonstrated that adjuvants could change droplet physicochemical properties, such as diameter and relative droplet span, thereby increasing the deposition amount on plant leaves and thus improving the utilization rate of pesticides (He et al., 2018). In addition, when the spray volume and droplet size are the same, the larger the droplet density, the higher the utilization rate of the chemical solution, and the better the control effect (Merritt, 1982). Combined with the results of contact angle and spreading rate, we found that adding spray adjuvants could increase the droplet size of the defoliant and reduce the risk of drift.

### Effect of aerial application of adjuvants on the droplet density sprayed by UAVs

**Figure 5:**
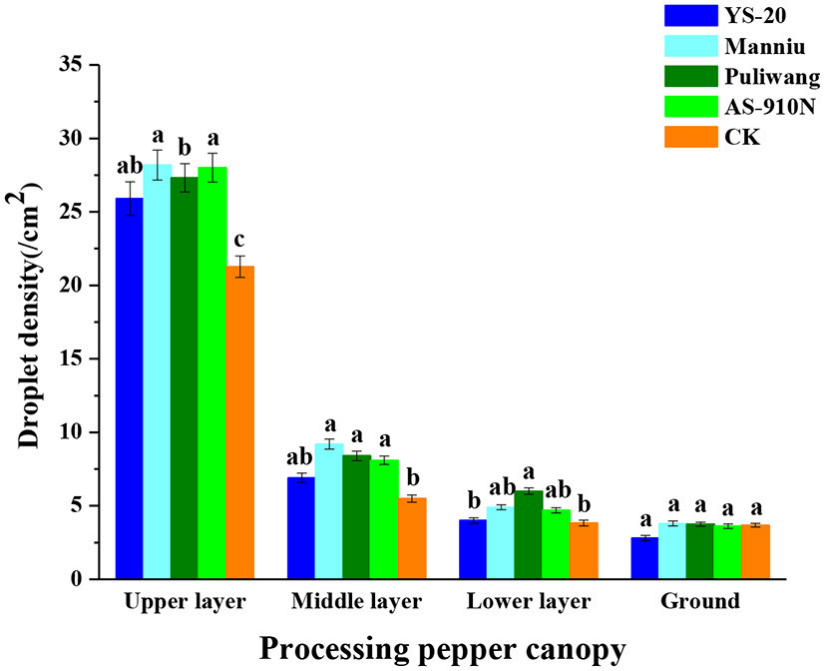
Effect of aerial spray with adjuvants on the droplet density sprayed by UAV. ^a−c^They represent the results of a significant difference analysis by Duncan's new multiple range test at the level of *P* < 0.05. Values followed by the same letter do not differ statistically.

The droplet density of defoliants varied greatly among the layers of the pepper canopy (Figure 5). Overall, the droplet density of the upper layer of the pepper canopy was higher than that of other layers, which was due to the interception of the defoliant droplets by the upper layer with a larger leaf area in the later growth stage of pepper. In the upper and middle layers of the canopy, the average droplet densities of adjuvant-added defoliants were 27.31 and 8.11/cm^2^ respectively, which were significantly higher than the CK (21.27 and 5.49/cm^2^), while in the lower layer and the ground, there was no significant difference. In addition, the droplet density in the upper layer with Manniu was the highest (28.18/cm^2^) among all the treatments, followed by AS-910N (28.01/cm^2^) and Puliwang (27.32/cm^2^), while the YS-20 (25.91/cm^2^) was significantly lower than others. The droplet density of the defoliant without adjuvants was significantly lower than the aerial application with adjuvants.

### Effect of aerial application with adjuvants on the droplet coverage sprayed by UAV

**Figure 6:**
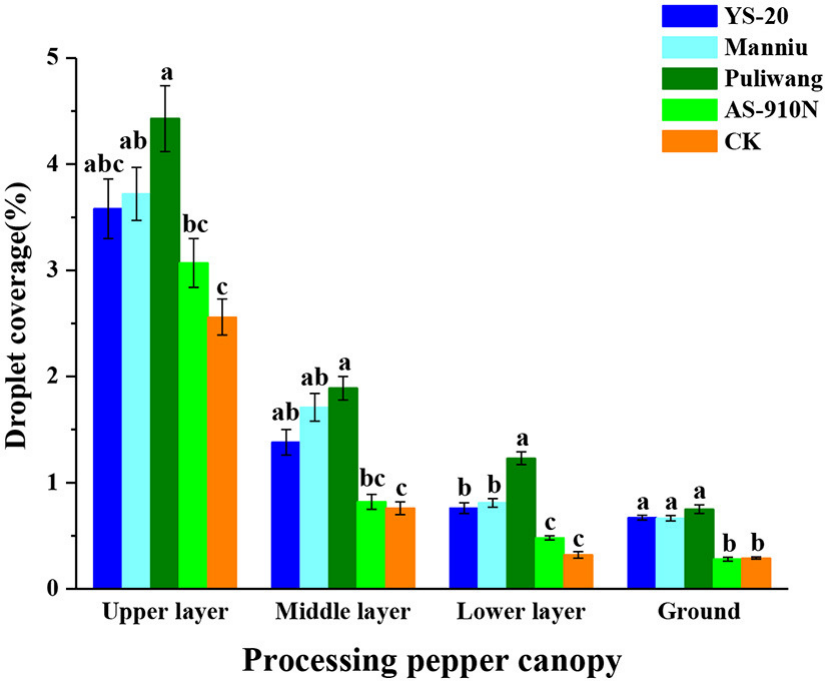
Effect of aerial spray with adjuvants on the droplet coverage sprayed by UAV. ^a−c^They represent the results of a significant difference analysis by Duncan's new multiple range test at the level of *P* < 0.05. Values followed by the same letter do not differ statistically.

**Figure 7:**
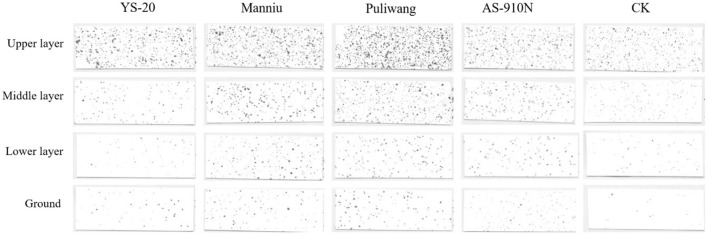
Effect of aerial spray with adjuvants on the droplet coverage sprayed by UAV (gray-scale photos of WSP).

Meng et al. (2020) found that adding adjuvants could increase droplet coverage of the canopy, which would increase the probability of the droplets hitting the target, thereby improving its efficacy (Meng et al., 2020). As the droplets are intercepted by the upper layer of the pepper canopy, the coverage rate of the upper layer was significantly higher than other layers (Figures 6, 7). The presence (or absence), and the type of adjuvant caused a significant impact on droplet coverage. The average droplet coverage in the upper, middle, and lower layers, and ground (3.46, 1.26, 0.7, and 0.51%) of the crops treated with added adjuvant was significantly higher than CK (2.56, 0.76, 0.32, and 0.29%). When Puliwang was added, the droplet coverage of the upper, middle, and lower layers, and ground (4.44, 1.84, 1.19, and 0.76%) was significantly higher than other adjuvants. Manniu and YS-20 had the second highest coverage, and AS-910N had the least, with no significant difference from CK. In general, the defoliant droplet coverage rate for the aerial spray with adjuvants was significantly higher than that of the control (Figure 6). Previous research has shown that influenced by the wind field of the UAV rotor, the defoliant droplet coverage rate, particularly the range of spray width, varies considerably (Li et al., 2018). It should be noted that in the upper layer of the pepper crops, the droplet coverage rate was significantly different, and the adjuvant-added treatments were significantly better than the control without any adjuvant. In the middle and lower layers and the ground, there were no such differences. This may be due to the influence of drift and evaporation on the deposition of droplets in the upper layer.

### Effect of aerial spray with adjuvants on the droplet uniformity sprayed by UAV

The uniform distribution of droplets is expressed by the coefficient of variation (CV) of the same canopy droplet deposition. The smaller the coefficient of variation, the better the uniformity of droplet distribution (Chen et al., 2021). The field experiments were influenced by environmental conditions and the CV of droplet density and coverage rate were relatively large. The average droplet distribution uniformity of the defoliant treated with Manniu was the best (60.65%), followed by YS-20 and Puliwang (66.14 and 66.47% respectively). The results of AS-910N were the poorest (83.65%), even inferior to the CK (82.26%) (Table 3). However, most of the droplets were deposited in the upper layer due to interception, so the distribution uniformity of the upper layer was more representative. The best uniformity of droplet distribution in the upper layer was Manniu (33.60%).

**Table 3:** Effect of aerial spray with adjuvants on the droplet uniformity sprayed by UAV.

**Treatment**	**Coefficient of variation (%)**			**Average**
	**Upper layer**	**Middle layer**	**Lower layer**	
YS-20	57.21	67.32	73.88	66.14
Manniu	33.60	72.50	75.84	60.65
Puliwang	56.83	70.32	72.25	66.47
AS-910N	60.97	90.59	99.39	83.65
CK	64.79	84.11	107.87	82.26

The uniformity of droplet distribution was measured by the CV of the deposition in the same canopy layer of peppers (Zhan et al., 2022). According to the Chinese Civil Aviation Industry Standard, in the case of low-volume spray operation, the quality of the operation can only be guaranteed when the coefficient of variation of droplet distribution is <60%. The average variation coefficients of the whole plant droplet distribution (66.14, 60.65, 66.47, 83.65, and 82.26%) in the current study seem to be not standard. The planting density of the peppers in the experimental field reached 213,000 plants/hm^2^ with an average height of about 0.88 m at the time of application. The interception effect of the upper layer was obvious, and its droplet variation coefficient (57.21, 33.60, 56.83, 60.97, and 64.79%) was more representative. Adding aerial spray adjuvants can reduce the coefficient of variation of droplet distribution which means improving the uniformity of droplet distribution, to meet the UAV operational standards.

### Effect of aerial applications with adjuvants on the droplet distribution sprayed by UAVs

**Figure 8:**
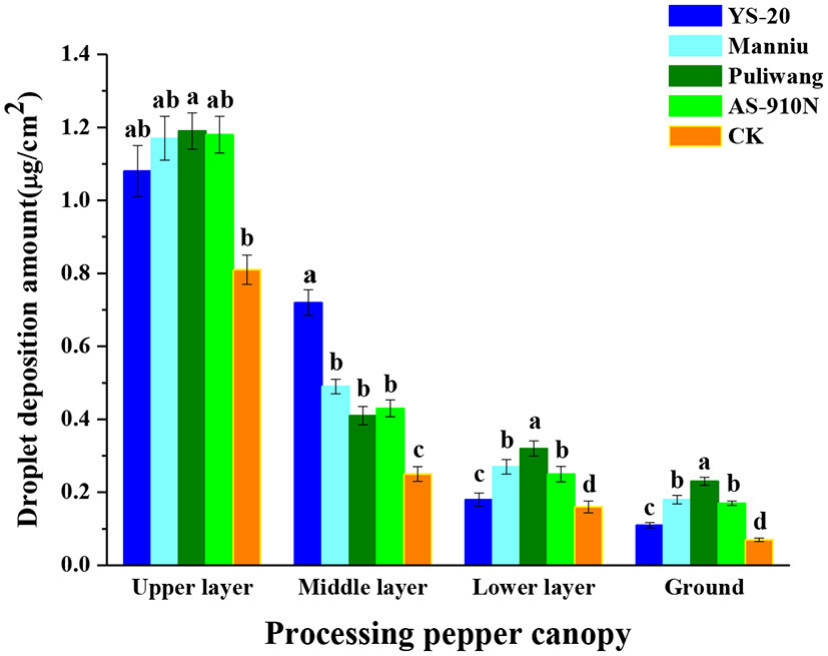
Effect of aerial spray with adjuvants on the droplet distribution sprayed by UAV. ^a−d^They represent the results of a significant difference analysis by Duncan's new multiple range test at the level of *P* < 0.05. Values followed by the same letter do not differ statistically.

The average deposition of droplets in the upper, middle, and lower layers and ground of the crops treated with adjuvants (1.15, 0.51, 0.25, 0.17 μg/cm^2^) was significantly higher than the CK (0.81, 0.25, 0.15, 0.07 μg/cm^2^) (Figure 8). In the upper layer, the droplet deposition amount of the Puliwang was the highest (1.19 μg/cm^2^). While in the middle layer, the YS-20 was the highest (0.72 μg/cm^2^). The droplet distribution penetration rates of YS-20, Manniu, Puliwang, and AS-910N were 15.51, 22.59, 27.11, and 20.97%, respectively, which were significantly higher than the CK (15.89%). The Puliwang showed a better effect in terms of penetration.

Some studies have suggested that droplets with smaller particle sizes are difficult to be intercepted by the upper layer and can penetrate better the middle and lower layers (Knoche, 1994; Wolf and Daggupati, 2009). Few other studies found that large particle-size droplets could not drift and evaporate easily and were more likely to reach the lower canopy layer (Derksen et al., 2008). In this study, we observed that the spraying penetration using Puliwang (27.11%) was better; it resulted in a larger droplet size and the DV50 reaching 402, 377, and 365 μm in the upper, middle, and lower layers. Therefore, in our study, penetration was better due to the larger droplet sizes.

### Effect of aerial spray with adjuvants on the deposition rate sprayed by UAV

The effective deposition rates (23.33, 21.08, 23.13, and 16.37%) of the four treatments with adjuvants were 5–15% higher than the CK (12.07%) with a significant difference (Table 4). In the upper layer of the pepper canopy, the effective deposition rate after adding Puliwang (39.3%) was the highest. In the middle layer, the YS-20 was the highest (23.2%). In the lower layer, there was no significant difference between the effective deposition rates of YS-20 and Puliwang (10.4 and 12.3%, respectively), while being significantly higher than the other treatments.

**Table 4:** Effect of aerial spray with adjuvants on the deposition rate sprayed by UAVs.

**Spraying date**	**Deposition rate (%)**			**Average**
	**Upper layer**	**Middle layer**	**Lower layer**	
YS-20	36.4 ± 6.1 ab	23.2 ± 2.5 a	10.4 ± 3.3 a	23.33
Manniu	35.7 ± 1.2 ab	21.4 ± 2.5 a	6.2 ± 2 b	21.08
Puliwang	39.3 ± 11.5 a	17.9 ± 3.6 b	12.3 ± 5.1 a	23.13
AS-910N	30.1 ± 5.1 ab	11.5 ± 0.3 c	7.5 ± 1.3 b	16.37
CK	25 ± 1.6 b	7.2 ± 1.2 d	4 ± 0.3 c	12.07

The data in the table are averages. Values followed by the same letter in the column do not differ statistically (p < 0.05).

The effective deposition rate of droplets could also be remarkably improved by adjuvants, because of the larger droplet size, the improved atomization effect, and the reduced evaporation and drift (Lan et al., 2008; Sijs and Bonn, 2020). The results of this study were similar to previous studies. In addition, adding adjuvants during pesticide spraying can change the physicochemical properties, promote the absorption of target plants or insects, and the retention of the liquid (Wang et al., 2022), thereby improving the utilization rate of pesticides.

### Effect of aerial sprays with adjuvants on the defoliation rate of pepper sprayed by UAV

The addition (and absence) of adjuvants had a significant effect on the defoliation rate of pepper (Table 5). Leaf abscission began to form three days after the first spraying and the aerial spray with adjuvants had a considerable effect on the defoliation effect. Three days after spraying, the defoliation rate of crops sprayed with adjuvants was higher than that of CK, and Puliwang showed the best defoliation effect (64.04%). Between 6 and 12 days after spraying, Puliwang still showed the best defoliation rate, but there was no significant difference among the four adjuvants. Fifteen days after spraying, the defoliation rate of Puliwang treatment was 98.40%, and that of YS-20 and Manniu was more than 95%. However, the defoliation rate of AS-910N was only 89.07% and that of CK was only 79.92%. The above results showed that the addition of adjuvants to aerial applications could significantly improve the defoliation rate of pepper, and the results obtained with the use of Puliwang were the best.

**Table 5:** Effect of aerial spray with adjuvants on the defoliation rate of processing pepper sprayed by UAVs.

**Days after spraying**	**Defoliation rate of processing pepper (%)**				**CK**
	**YS-20**	**Manniu**	**Puliwang**	**AS-910N**	
3	46.14 abc	47.24 ab	64.04 a	36.55 bc	20.30c
6	61.40 ab	68.33 ab	79.07 a	53.13 ab	42.31b
9	75.74 ab	75.06 ab	88.40 a	70.32 ab	62.55b
12	85.63 ab	83.19 ab	92.84 a	78.86 ab	73.04b
15	95.21 ab	95.58 ab	98.40 a	89.07 b	79.92c

The data in the table are averages. Values followed by the same letter in the column do not differ statistically (p < 0.05).

### Effect of aerial sprays using adjuvants on the yield of pepper sprayed by UAVs

The effects of adding adjuvants to aerial applications on the yield of pepper are shown in Table 6. The average yield of pepper using adjuvants (518.00 kg/666.7 m^2^) was significantly higher than that of the CK (446.85 kg/666.7 m^2^). The yield of adding Puliwang (540.19 kg/666.7 m^2^) was slightly lower than Manniu (543.41 kg/666.7 m^2^), but significantly higher than other treatments. Puliwang and Manniu could significantly improve the yield of peppers; their yield increase rate exceeded 20%.

**Table 6:** Effect of aerial sprays using adjuvants on the yield of pepper sprayed by UAVs.

**Treatment**	**Fresh weight per 15 plants** **(g)**	**Dry weight per 15 plants** **(g)**	**DW/ FW ratio** **(%)**	**Theoretical yield of fresh pepper** **(kg/667 m^2^)**	**Theoretical yield of dry pepper** **(kg/667 m^2^)**	**Increase of dry pepper** **(%)**
Puliwang	936.33	671.33	71.69	753.43	540.20 a	20.89
Manniu	928.67	675.33	72.72	747.27	543.42 a	21.61
YS-20	921.67	620.67	67.34	741.64	499.43 b	11.76
AS-910N	1174.33	608.00	51.77	944.94	489.24 b	9.48
CK	799.67	555.33	69.44	643.47	446.86 c	/

The data in the table are averages. Values followed by the same letter in the column do not differ statistically (p < 0.05).

**Figure 9:**
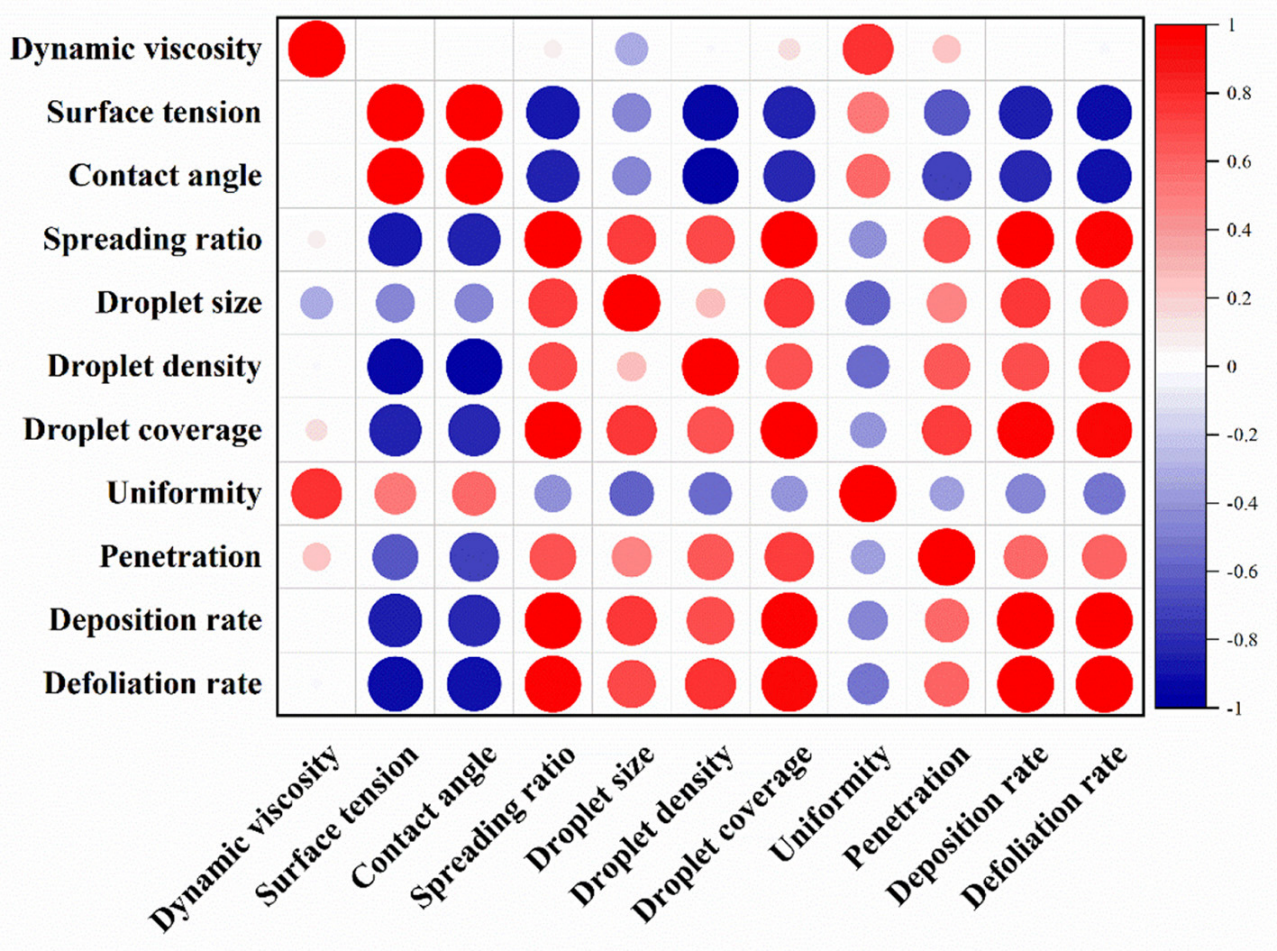
Heat map for the pearson correlation coefficients. The color and size of the circles represent the *r* and *p-*values. Deep and large circle signal a significant correlation.

To sum up, the aerial applications using adjuvants had varying degrees of effects on the physicochemical properties, droplet deposition, and defoliation rate of the pesticide solution (Table 7). The performance of adjuvants could be evaluated based on these effects as indicators. Pearson correlation analysis was used to study the relationship between these indicators (Figure 9). The results showed that surface tension was significantly positively correlated with contact angle (*r* = 0.987, *p* < 0.01), and significantly negatively correlated with spreading ratio and defoliation rate (*r* = −0.883 and −0.937, *p* < 0.05). This indicates that the addition of adjuvants could effectively reduce the surface tension, thereby promoting the spreading of the droplets. In addition, the spreading rate was significantly positively correlated with droplet coverage (*r* = 0.989, *p* < 0.01), deposition rate (*r* = 0.992, *p* < 0.05), and defoliation rate (*r* = 0.980, *p* < 0.05). The droplet coverage was also significantly positively correlated with deposition rate (*r* = 0.966, *p* < 0.01) and defoliation rate (*r* = 0.946, *p* < 0.05). It could be seen that the adjuvants improved the spreading ratio of the droplets, the coverage rate, and the deposition rate, so that the contact between the defoliant and the pepper leaves was increased, which finally enhanced the defoliation effect. Furthermore, although not significant, there was a positive correlation between droplet size and droplet distribution penetration (*r* = 0.494), which supports the previous observation. In addition, the dynamic viscosity had a certain effect on the droplet size (*r* = −0.335). Specifically, the higher the viscosity, the smaller the droplets produced by the UAV spray, which was consistent with previous studies (Jamalabadi et al., 2017).

**Table 7:** The effects of aerial application of adjuvant-enhanced defoliants on physicochemical properties, droplets deposition, and defoliation rate.

**Adjuvants**	**Physicochemical properties**				**Droplet deposition**						**Defoliation rate (%)**
	**Dynamic viscosity** **(mPa/s)**	**Surface tension** **(mN/m)**	**Contact angle** **(°)**	**Spreading ratio** **(%)**	**Droplet size** **(μm)**	**Droplet density** **(/cm^2^)**	**Droplet coverage** **(%)**	**Uniformity** **(%)**	**Penetration** **(%)**	**Deposition rate** **(%)**	
YS-20	1.24	45.83	44.28	35.35	373	25.91	3.61	57.21	15.51	36.4	95.21
Manniu	1.08	43.47	31.32	34.54	397	28.18	3.70	33.60	22.59	35.7	95.58
Puliwang	1.37	42.57	31.34	46.21	402	27.32	4.44	56.83	27.11	39.3	98.4
AS-910N	1.34	45.73	38.56	24.17	320	28.01	3.15	60.97	20.97	30.1	89.07
CK	1.25	60.47	73.40	10.81	348	21.27	2.56	64.79	15.89	25.0	79.92

**Figure 10:**
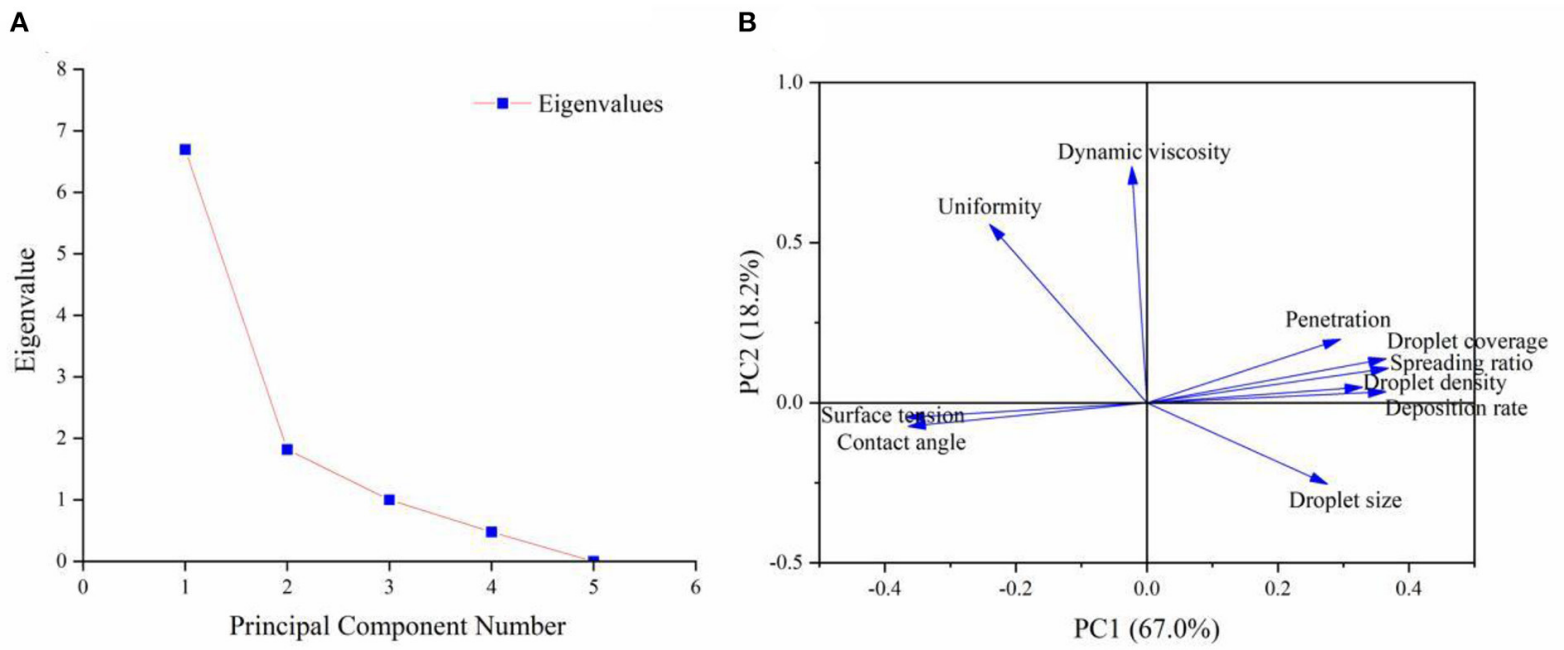
Scree plot of eigenvalues for principal components **(A)** and loading plot for principal components 1 and 2 **(B)**.

Through the correlation analysis, we found that the correlation between the indicators caused obstacles to the comprehensive evaluation of different adjuvants. Therefore, principal component analysis (PCA) was used to comprehensively evaluate the adjuvants. We selected two principal components whose cumulative contribution rate of eigenvalue reached 85.16% (Figure 10A). The variance contribution rates of principal components 1 and 2 were 66.97 and 18.19% respectively, indicating that it could effectively reflect the original data in the auxiliary indicators. The loading plot for principal components was used to measure the contributions of the principal components. Specifically, a larger absolute value of the load means that the contribution of the corresponding principal component is larger (Karytsas and Choropanitis, 2017). Principal component 1 had a large to small load in terms of spreading ratio, contact angle, droplet coverage, surface tension, deposition rate, droplet density, penetration, and droplet size (Figure 10B). Principal component 2 had a large load in terms of uniformity and dynamic viscosity (Figure 10B). These results showed that except for uniformity and dynamic viscosity, other indicators could reflect the performance of the aerial application of adjuvants to a large extent, especially spreading ratio, contact angle, droplet coverage, surface tension, deposition rate, and droplet density.

Based on the mathematical model of PCA, we found that the comprehensive score for evaluating the performance of adjuvants (Table 8). Puliwang had the highest comprehensive score among the four adjuvants, followed by AS-910N, YS-20, and Manniu. The score of CK without additives was only 1.1806, far lower than the other four treatments. Therefore, it can be established that Puliwang had the best performance.

**Table 8:** Comprehensive score of adjuvant performance.

**Adjuvant**	**Scores of first principal components (F1)**	**Scores of second principal components (F2)**	**Comprehensive score (F)**
YS-20	0.0303	−0.1078	0.0008
Manniu	0.3163	−1.6467	−0.1023
Puliwang	1.2190	0.6833	1.1046
AS-910N	−0.0123	0.8793	0.1781
CK	−1.5533	0.191966.	−1.1806

## Conclusion

In this study, pepper processing and aerial spray adjuvants were selected as research objects, and the type of adjuvant that could effectively improve the defoliation effect of the pepper when sprayed by UAV was determined. Specifically, we studied the effects of aerial spray adjuvants on the physicochemical properties of the pepper defoliants. On that basis, the effects of various adjuvants on droplet deposition and defoliation of pepper crops were determined by spraying adjuvant enhanced defoliants using UAVs. The results of correlation analysis and principal component analysis show that Puliwang had the best effect as an adjuvant for aerial application of defoliants.

## Data availability statement

The original contributions presented in the study are included in the article/supplementary material, further inquiries can be directed to the corresponding author.

## Author contributions

XH conceived and designed the experiments and wrote the paper. YL, QX, ZF, and ZD performed the field experiments. YL, QX, and XH analyzed the data. MZ conceived the research and revised the manuscript. All authors contributed to the article and approved the submitted version.

## Funding

This research was funded by Xinjiang Production and Construction Corps Major Scientific and Technological Projects, grant number 2018AA010-03 and the National Key Research and Development Program of China, grant number 2016YFD0200700.

## Conflict of interest

The authors declare that the research was conducted in the absence of any commercial or financial relationships that could be construed as a potential conflict of interest.

## Publisher's note

All claims expressed in this article are solely those of the authors and do not necessarily represent those of their affiliated organizations, or those of the publisher, the editors and the reviewers. Any product that may be evaluated in this article, or claim that may be made by its manufacturer, is not guaranteed or endorsed by the publisher.
